# Acknowledging the peer reviewers of *Journal of Medical Radiation Sciences*, October 2022 – September 2023

**DOI:** 10.1002/jmrs.732

**Published:** 2023-11-23

The editorial review board, the Australian Society of Medical Imaging and Radiation Therapy (ASMIRT) and the New Zealand Institute of Medical Radiation Technology (NZIMRT) acknowledge the following peer reviewers for their dedication and commitment to the journal. Thank you for sharing your valuable time and knowledge.

## Outstanding Reviewers (Contributed to Five Reviews)

Ann Poulos (eight reviews)

James Stanley (seven reviews)

Marilyn Baird

Georgia Halkett

## The Following Reviewers Contributed to Four Reviews

Lynne Hazell

Peter Hogg

Andrew Kilgour

Dean Paterson

## The Following Reviewers Contributed to Three Reviews

Kamarul Abdullah

Vikneswary Batumalai

James Crowhurst

Paul Kane

Shivani Kumar

Yu‐Feng Wang

Doaa Elwadia

## The Following Reviewers Contributed to Two Reviews

Dania Abu Awwad

Nigel Anderson

Sally Ayesa

Cameron Brown

Glenn Cahoon

Beini Chen

Jenna Dean

Nicolas Depauw

Laura di Michelle

Ernest Ekpo

Kelly Elsner

Andrew England

Andrew Firman

Hesta Friedrich‐Nel

Robin Hart

Bronwyn Hilder

Rebecca Jude

Aidan Leong

Jens Loberg

Chandra Makanjee

Sibusiso Mdletshe

Andrew Murphy

Vanessa Panettier.

Tracey Pieterse

Jonathan Loui Portelli

Robba Rai

Clare Singh

Tom Steffens

Kaiwen Xu

Don Nocum

## The Following Reviewers Contributed to One Review

Steven Abbott

Mohamed Abuzaid

Joanne Adlam

Dana S. Al‐Mousa

Fahim Alam

Mohammad Aljamal

Alomaim, Wijdan

Alphonse, Jennifer

Alsleem, Haney

Elio Arruzza

Murat Baykara

Rachael Beldham‐Collins

Jemma Blyth

Eeva Boman

Vicki Braithwaite

Remco de Bree

Jerome Breitenberger

Philippa Bresser

Bena Brown

Elizabeth Brown

Teresa Brown

Melissa Burns

Michael Cardoso

Nahid Chegeni

Jasmine Chen

Deanne Chester

Phillip Chlap

Benjamin Chua

Emma Cooper

Ben Daniel

Pernilla Darlington

Yves De Deene

Aimee Devlin

Karen Dobeli

Edel Doyle

Kylie Dundas

Gay Dungey

Ernest Eduful

Christopher Edwards

Mel Evans

Melissa Ferguson

John Fernandez

Veronica Ferrero

Chantelle Fisher

Rhys Fitzgerald

Sheryl Foster

Ziba Gandomkar

Vivien Gibbs

Peter Gorayski

Clinton Gould

Therese Gunn

Gaorav Gupta

Catriona Hargrave

Amir Human Hoveidaei

Felicity Hudson

Michael Huo

Brian F. Hutton

Emma Hyde

Alannah Kejda

Peter Kench

John Kenny

Laurence Kim

Min Ku

Kathryn Lamb

Heather Lawrence

Kelly Lloyd

Neil Lunt

Sharon Maresse

Paul Marks

Harry Marquis

Hamidreza Masjedi

Brianna McCoola

John McInerney

Clare McKenzie

Helen McNair

Bronwen Merner

Renee Mineo

Martin Mitchell

Rahul Modi

Hanns Moshammer

Shyamsundar Muthuramalingam

Glen Newman

Michelle O'Connor

Peter O'Reilly

Donna Oomens

Eric Pei Ping Pang

Scott Penfold

Mario Perez

Natalie Pollard

Ayyaz Qadir

Clare Rainey

Albertina Rusandu

Catherine Russell

Susan Said

Daniel Scandurra

Benjamin Searle

Hongming Shan

Meegan Shepherd

Kiarash shirbandi

Bronwyn Shirley

Kate Skehan

Edward Smith

Rachel Stensmyr

Adam Steward

Kate Stewart

Kristie Sweeney

Samantha Thomas

John Thompson

Jacqueline Veera

David Walton

Mark West

Alison White

Imelda Williams

Bridget Wyrley‐Birch

Karim Yacoub

Adam Yeo

Adrienne Young

Maria Zankl

Xiaoming Zheng

Peter Greer

Brendan Erskine

Tarni Nelson

## Peer Reviewer Resources

Are you new to conducting and writing peer reviews? Check out these free peer reviewer guidelines and self‐directed learning modules.

- Wiley Journal Reviewers

https://authorservices.wiley.com/Reviewers/journal-reviewers/index.html

b Wiley Researcher Academy: Becoming a Peer Reviewer

https://upskillstudio.researcher.life/p/wiley-researcher-academy-becoming-a-peer-reviewer

c Web of Science Academy

https://clarivate.com/webofsciencegroup/solutions/web-of-science-academy/

## Peer Reviewer Recognition

JMRS reviewers can opt in to have their peer review contribution automatically added on Web of Science Reviewer Recognition Services (formerly Publons).

For more information please go to, https://authorservices.wiley.com/Reviewers/journal-reviewers/recognition-for-reviewers/publons.html

